# Unilateral Heliotrope Rash in Juvenile Dermatomyositis: An Unusual Presentation of an Underlying Serious Disease

**DOI:** 10.1155/2014/979856

**Published:** 2014-12-22

**Authors:** Ghada Al-Janobi, Hisham Alkhalidi, Mohammed A. Omair

**Affiliations:** ^1^Division of Rheumatology, Department of Medicine, College of Medicine, King Khalid University Hospital, King Saud University, P.O. Box 2925, Riyadh 11461, Saudi Arabia; ^2^Division of Rheumatology, Department of Medicine, Qatif Central Hospital, Qatif, Saudi Arabia; ^3^Department of Pathology, College of Medicine, King Saud University, Riyadh, Saudi Arabia

## Abstract

*Background*. Heliotrope rash is one of the characteristic skin manifestations of juvenile dermatomyositis. It is a reddish-purple rash on the upper eyelids that is usually bilateral. *Case Presentation*. We report a boy who presented with unilateral heliotrope rash, Gottron's papules, and muscle weakness. Muscle biopsy was consistent with inflammatory myositis. Patient was started on prednisolone and methotrexate with an excellent response in both the skin and muscles. *Conclusion*. Unilateral heliotrope rash can occur in patients with juvenile dermatomyositis. Being a paraneoplastic condition caution should be taken not to miss any underlying malignancy.

## 1. Background

Juvenile dermatomyositis is a multisystem diseases characterized by vasculopathy of the skin and/or muscles causing symmetrical proximal weakness and typical skin rashes [1]. Heliotrope rash is a reddish-purple rash on the upper eyelids, often accompanied by swelling of the eyelid [2]. It occurs in up to 86.7% of cases [3] and is usually bilateral. Here we report a 14-year-old boy presenting with unilateral heliotrope rash and muscle weakness.

## 2. Case Presentation

A 14-year-old previously healthy boy presented with left periorbital swelling and redness for 1 year and muscle weakness and joint pain for 4 months.

**Figure 1:**
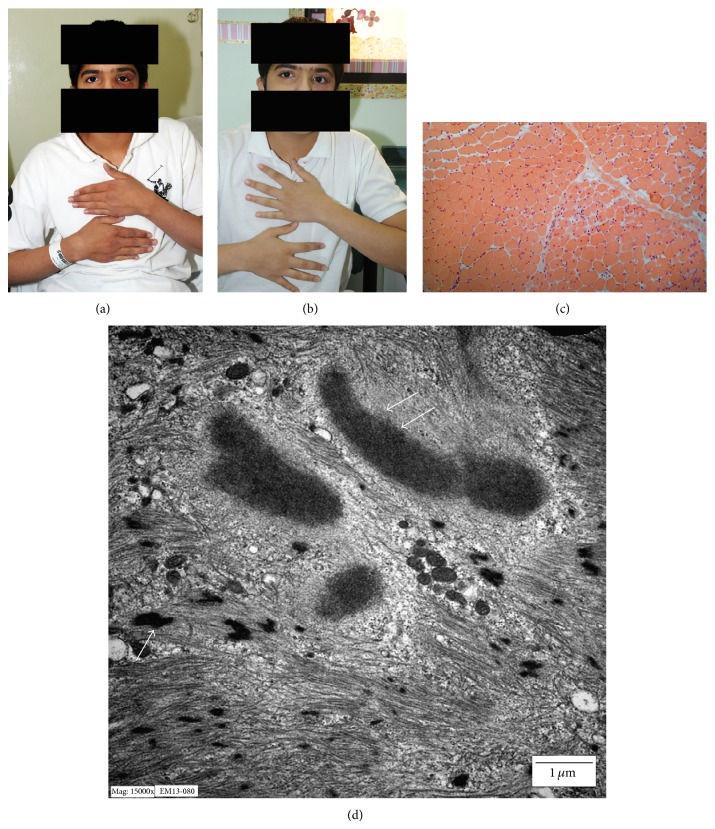
((a) and (b)) Appearance of the unilateral heliotrope rash along with Gottron's papules before and after 6 months of therapy, respectively. (c) Focal perifascicular atrophy was focally appreciated on light microscopy (H&E, ×100). (d) Ultrastructural examination revealed areas with prominent myofibrillar disarray with scattered rod-like structures (single arrow) and cytoplasmic bodies (double arrows) (×15000).

On examination he had purple discoloration and swelling around the left eye (Figure 1(a) before therapy and Figure 1(b) after therapy), Gottron's papules over the metacarpophalangeal (MCP) joints bilaterally, and muscle weakness with a grade of 3/5 in the proximal group and 4/5 in the distal group. Workup revealed a normal CBC, ESR 33 mm/hr, and CRP 0.383 mg/L. Liver function test showed the following abnormalities: AST 281 U/L, ALT 95 U/L, GGT 24 U/L, ALP 149 U/L, and CK 4585 U/L. Antinuclear antibodies negative with ENA are all negative. Renal function test and thyroid function test were normal.

MRI muscle revealed diffuse muscle edema involving the muscles of the pelvis, thighs, legs, and upper extremities as well as the muscle of back suggestive of inflammatory myopathy.

CT orbital shows soft tissue swelling in the anteromedial and superior aspects of the left orbit which shows minimal enhancement in the postcontrast, there was an appearance of a left-sided preseptal periorbital cellulitis with no evidence of abscess formation. Biopsy from the lower lid revealed no malignant cells or acid fast bacilli with subsequent negative culture for tuberculosis after 8 weeks.

Muscle biopsy was performed and revealed mildly and focally increased endomysial and perimysial connective tissues. There was perivascular chronic inflammatory cells infiltration in the perimysial areas with few mononuclear inflammatory cells that were scattered in-between the muscle fibers. The muscle fibers showed mild to focally moderate variation of size and shapes and the majority had peripheral nuclei. Scattered foci of myofiber necrosis and regeneration were evident. Perifascicular atrophy was not a prominent feature, but it could be focally appreciated (Figure 1(c)). Ultrastructural examination revealed findings that were in keeping with light microscopy, including prominent myofibrillar disarray (Figure 1(d)). In addition, scattered rod-like structures and cytoplasmic bodies were detected. The overall features were in keeping with an inflammatory myopathy, with features suggestive of dermatomyositis.

Skin biopsy of the left lower lid showed evidence of mild hyperkeratosis in the epidermis. The dermis showed heavy chronic inflammation cell infiltration that consists mainly of lymphocytes and plasma cells, infiltrating the hair follicles. Adjacent mild dermal fibrosis, focal solar elastosis, and pigment incontinence are noted.

The patient was started on prednisolone 50 mg daily with an increasing dose of methotrexate 15 mg reaching 20 mg per week. At 6 months, the patient showed a dramatic improvement with normalization of muscle power, fading of the skin rashes, and reduction of muscle enzymes. As of April 2014, he is back to school with a normal performance on prednisolone 5 mg daily and methotrexate 20 mg weekly.

## 3. Discussion

When evaluating a patient with JDM with unilateral periorbital swelling, caution should be taken not to miss an infectious etiology or infiltrative malignancy which is a well-recognized association [4]. Imaging and biopsy of the periorbital swelling are warranted before starting immunosuppressive therapy. Despite the fact that our patient's treatment was naive, we did not see the full features of JDM in the biopsy. Additionally, electronic microscopy revealed scattered rod-like structures and cytoplasmic bodies which are not commonly seen but previously described in JDM [5, 6]. Treatment of cutaneous manifestations of JDM includes photoprotection, topical corticosteroids, topical calcineurin inhibitors, and antimalarials as first line. Second line agents include corticosteroids, methotrexate, and mycophenolate mofetil. In refractory cases drugs such as dapsone, azathioprine, intravenous immunoglobulins, and rituximab can be tried [7]. In our case, the response to corticosteroids and methotrexate was adequate for both muscle and skin manifestations. To our knowledge, this is the first case presenting with a unilateral heliotrope rash and successful treatment with standard immunosuppressive agents.
